# Use of European Funds and Ex Post Evaluation of Prevention Measures against Wolf Attacks (*Canis lupus italicus*) in the Emilia-Romagna Region (Italy)

**DOI:** 10.3390/ani11061536

**Published:** 2021-05-25

**Authors:** Duccio Berzi, Jacopo Cerri, Carmela Musto, Maria Luisa Zanni

**Affiliations:** 1 Ischetus scrl, Viale Ugo Bassi 6/r, 50137 Florence, Italy; 2 Faculty of Mathematics, Natural Sciences and Information Technologies, University of Primorska, Glagoljaška 8, 6000 Koper, Slovenia; jacopocerri@gmail.com; 3 Department of Veterinary Medical Sciences, University of Bologna, 50 Via Tolara di Sopra,40064 Ozzano dell’Emilia (BO), Italy; carmela.musto2@unibo.it; 4 Pianificazione e Osservatorio Faunistico, Regione Emilia-Romagna, Viale della Fiera 8, 40127 Bologna, Italy; MariaLuisa.Zanni@regione.emilia-romagna.it

**Keywords:** Italian wolf, wolf–livestock conflicts, prevention programs, European funds, coexistence, wildlife strategies, wildlife economy

## Abstract

**Simple Summary:**

Starting in 2014 in a region in Northern Italy, Emilia-Romagna, a specific pilot project was financed through regional budget resources, aimed at spreading wolf damages’ prevention measures, accompanied by a technical assistance program for the breeders requesting it. In particular, standardized types of intervention were defined, inspections were organized of the damaged companies, and technical assistance was structured. Ex post, the effectiveness of the interventions and the degree of satisfaction were assessed. The pilot project financed with regional budget funds was then accompanied by a call from the European Agricultural Fund for Rural Development and subsequent interventions were financed with other regional budget funds. This contribution analyzes the difficulties encountered in using the various prevention tools, the effectiveness of the operation of the mitigation measures, and the degree of user satisfaction.

**Abstract:**

Introduction: Compensation programs are an important tool for mitigating conflicts between farmers and large predators. However, they present significant weaknesses and faults. For years, the EU has been prioritizing programs for the prevention of damage caused by large carnivores, rather than compensation programs, introducing compulsory compensation for the purposes of decision EC (2019) 772 of 29/01/19. This manuscript reports the experience with the wolf damage prevention programs in an Italian region, Emilia-Romagna, which implemented a pilot project, adopting a new method to interface with the farmers involved in the prevention programs. Methods: Starting in 2014, a project aimed at spreading prevention measures was financed through regional and European resources, accompanied by resources sharing and technical assistance with breeders from the regional body. In detail, (i) standardized types of intervention were defined and technical assistance was structured; (ii) ex post, the effectiveness of the interventions carried out was assessed; and (iii) the difficulties encountered in using the various financing instruments were analyzed. Results: Overall, 298 farms were analyzed, of which 166 applied for regional calls and 132 applied for European funds. The mitigation measures produced a reduction in predatory phenomena of 93.4%, i.e., from 528 to 35 predations over a period of 4–6 years. This study shows that more than one-third of the farmers were forced to abandon the two tenders, mainly due to the lack of liquidity in anticipating the prevention measures. Conclusion: In the years examined by this study, the prevention programs in the Emilia-Romagna region, due to the technical support offered, proved to be a functional and effective tool, capable of significantly reducing the wolf predation on livestock. However, this work highlights the high percentage of denials of mitigation measures by farmers interested in adopting these tools, stressing the need for regional agencies to focus on new policies that can provide advance economic resources to farmers and solve the authorization problems related to the various bodies with which the participant in the tenders must interface.

## 1. Introduction

The reasons for the success of large carnivores in Europe range across the coordinated legislation shared by many European countries, context-specific management practices, and institutional arrangements [1]. However, the positive trend in carnivore populations—such in the case of grey wolves, which have managed to recolonize areas where they had long been absent—has increased the human–predator conflict due to the damage to livestock [2,3,4].

To mitigate this human–carnivore conflict, since the 1970s, various compensation programs have been adopted, providing repayment for livestock killed or injured by iconic species, e.g., wolves (*Canis lupus*) [5], brown bears (*Ursus arctos*), or Eurasian lynx (*Lynx lynx*) [6]. These compensation programs aim to offset the costs of having and sharing the landscape with large carnivores, reimbursing those who have suffered attacks on livestock [7]. In most countries, the compensation is paid retrospectively, based on the damage assessment. Only the Swedish authorities implement a different approach for reindeer, paying the Sámi reindeer herders beforehand (ex ante compensation), based on the estimated abundance or reproduction of large carnivores, regardless of the amount of the economic losses [8]. In Europe, the annual compensation for the damage due to large carnivores is approximately EUR 28.5 million [6], although the methods of assessing the damage are not harmonized even at a national level [5] and many administrations are in default of their duties toward the affected category.

Compensation programs are expected to alleviate conflict by increasing the tolerance of the presence of carnivores and by shifting economic costs toward the community [7]. For a short period, wildlife damage payments can generate local support for conservation, reduce the incentives for retaliatory actions [9,10], and buy time for alternative management practices [11], but these effects do not seem to last long. According to Nyhus et al. [12], the characteristics of a successful compensation scheme are: (1) quick and accurate verification of the damage; (2) prompt and fair payments; (3) long-term sustainable funding; (4) specificity of the site; (5) clear rules and guidelines; (6) a final assessment of its effectiveness. However, these conditions are difficult to achieve, mainly due to late payments [13], the absence of specialized figures in environmental agencies [7], the lack of coordination and available data [5], and a bureaucracy that is often too detached from the real local needs. Another problem related to the use of compensation programs, in the absence of any control, is that some livestock owners may reduce their protection efforts or may deliberately inflate losses from large carnivores [13,14].

Although compensation schemes are supposed to improve attitudes and tolerance toward large carnivores, thereby decreasing their persecution, available evidence indicates that this is not always the case [5,6,13,15,16]. Therefore, compensation systems are unlikely to be the ultimate solution unless combined with measures that effectively reduce the risk of harm [17]. An integrated risk management plan should involve both measures to prevent harm and incentives to compensate damages [13,18].

To mitigate conflicts and optimize the cost-effectiveness of publicly funded measures, the responsible agencies should be proactive, focus on prevention-based policies, and periodically assess the effectiveness of the compensation and prevention programs in an adaptive way [6].

Based on available scientific evidence of the effectiveness of mitigation measures, the European Commission invited its member states to condition reimbursement policies for large carnivores, upon the adoption of “adequate prevention measures” [19,20]. These include livestock guardian dogs (LGDs), night confinement of livestock, surveillance, fencing, or a combination of them, which was found to have the highest effectiveness [21], especially when followed by ex post monitoring [22]. Many regional administrations in member states, such as Italy, aligned with this policy. For example, the Emilia-Romagna region financed interventions through the de minimis regime (articles 107–108 of the Treaty on the Functioning of the European Union (TFEU)) or with resources from the European Agricultural Fund for Rural Development (EAFRD) through Rural Development Programs (RDPs).

The use of these resources, up to 100% of the eligible expenditure, appears to be a good opportunity for local authorities, but it uses procedures that need to be analyzed to avoid funding unnecessary interventions and to reduce cases of breeders not taking full advantage of the initiative due to a demanding bureaucracy. Moreover, there are relatively few evaluations of the effectiveness of the interventions, because this type of intervention is frequently applied within the context of local projects and in limited numbers, and because they are often executed extemporaneously by individual companies without any real technical standardization. In a context in which European environmental policies aim for the conservation of predators on a large scale [1] and in which the possibility of compensating for the killed animals is bound to the adoption of prevention measures [19,20], it is expected that many local administrations will intensify projects to spread prevention works. As such, large-scale studies of the ex post effectiveness of the interventions are hoped to become the practice for any project. Moreover, we wish for the issues regarding the management of the EAFRD to be considered in future planning.

The aims of the study were: (i) summarizing the difficulties encountered by farmers in using the various financing instruments; (ii) analyzing the effectiveness of the prevention programs funded and the degree of user satisfaction; (iii) illustrating how an adequate management on small scale can play a key role in the human–predator coexistence process.

## 2. Materials and Methods

### 2.1. Study Area and Adopted Mitigation Measures

**Figure 1:**
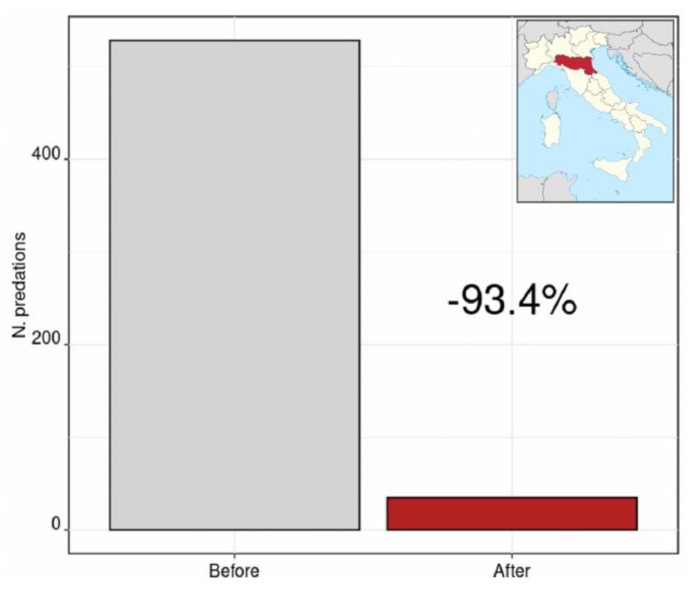
Overall number of predatory events on monitored farms before and after the adoption of mitigation measures. At the top right, the map shows the Italian region, Emilia-Romagna, the subject of this study, in red.

In this study, we focused on the Emilia-Romagna region (Figure 1), an area of Northern Italy that, in recent decades, faced a recovery and a geographical expansion of population of the Italian wolf (*Canis lupus italicus*) [23,24,25,26]. This region, as well as others in Italy, adopted compensation procedures for the damage caused by wolves, immediately after ensuring the species’ legal protection throughout the national territory [5]. The Emilia-Romagna region has an extensive hilly area where numerous agricultural activities with cattle breeding have arisen. In these hilly and lowland areas, the wolves were absent for decades and their return generated episodes of predation on livestock, with a growing increase in the amounts of compensation paid by the region to farmers, rising to the point of becoming economically impacting and politically unpopular [27].

During 2014–2015, the Emilia-Romagna region developed a pilot tender funded with budgetary resources (hereinafter called ERR) with a first total amount of EUR 425,000, accompanied by the implementation of interventions (Figures S1 and S2; Scheme S1), technical assistance, and sharing of the choices with the farmers. The technical assistance consisted of a visit by a technician during the intervention selection phase, assistance during the construction phase, and finally, the intervention was tested.

The call provided for 100% coverage of material purchase costs and up to a maximum of EUR 4000 per intervention, disbursed under the de minimis regime [28]. This call for proposals was also used in subsequent years to finance interventions limited in economic size.

The ERR regional call was followed for the year 2016 by the European EAFRD call (type of operation 4.4.02, “Prevention of damage from wildlife” [29]), aimed at financing wildlife prevention interventions, with a total economic availability of EUR 3,011,550. The extent of the interventions was between EUR 3000 and 30,000 and 100% financed, including humanpower.

The works financed by the calls were chosen based on experiments conducted in other contexts [30] and through a tailor-made approach chosen on the basis of each context analyzed. It was important to set up the interventions adapting them to every single circumstance, thus avoiding top-down approaches.

The different types of intervention implemented in this work are shown in supplementary materials Figures S1 and S2, and Scheme S1.

### 2.2. Data Collection and Statistical Analysis

To conduct the investigation on the issues related to the use of funds for the prevention of wolf damage, in this study, we analyzed both: (i) data about expenditures and damage, provided by the regional offices (the STACP, Italian acronym for “Territorial Service for agriculture, hunting and fishing”); and (ii) data from in-depth telephone interviews with the farmers about the outcomes of mitigation measures, predatory events before and after the implementation of measures, and the degree of satisfaction.

Regional data included the overall number of farms that applied for funding initiatives under the various programs, the number of mitigation measures that had been implemented, as well as the number of farms that dropped out from prevention programs, despite having been declared eligible for funding. Subsequently, information was collected on the reasons driving farmers to drop out from funding mechanisms, despite being eligible.

Interviews with farmers were conducted by a trained technician, following a semi-structured protocol. At first, farmers were reminded that the results from the interviews would be confidential, then the operator talked with farmers about adopted measures, eliciting their opinions about: (i) their overall satisfaction, (ii) effectiveness in reducing predations, (iii) cost of realization, and (iv) maintenance commitment/cost. Open-ended answers were categorized dichotomously, with farmers being deemed to have provided an affirmative answer only when they clearly stated that measures were effective/economically viable or affordable. Uncertain answers were treated as negative to make conservative estimates in statistical modeling.

Predations were assessed by a regular monitoring of farms carried out by field technicians in the 36 months before the implementation of prevention measures and for variable timespans after their adoption (6 months, *n* = 31; 12 months, *n* = 38; 18 months, *n* = 12; 24 months, *n* = 67; 30 months, *n* = 33; and 36 months, *n* = 38).

Then, we modeled how the perceived and real effectiveness of interventions drove farmers’ satisfaction with mitigation measures using Bayesian logistic regression [31], where the probability of a farmer being satisfied with the adopted measures was modeled as a function of whether they believed the intervention: (i) effective at reducing predations, (ii) economically viable, and (iii) feasible in its maintenance. We also added two dichotomous predictors, indicating if respondents had suffered any predation before and after the mitigation measure, to account for the effect of real damage. We pooled answers from farmers who benefited from different mitigation measures because, for some of them, we had few counts, and because the allocation of measures was not a random process and could have reflected a pre-existing heterogeneity in farming conditions.

We checked for collinearity in model covariates, with the variance inflation factor (VIF). Our model was fit with STAN [32] and it had 4 MCMCs with 5000 iterations each; we inspected model convergence and carried out posterior predictive checks to assess model fitness to the data. To increase model regularization, we adopted weakly informative prior distributions for slope parameters, specifying them as a normal distribution with mean equal to zero and variance equal to 1 [33]. Model selection was based on a back-wise approach, retaining significant covariates based on leave-one-out cross validation [34].

## 3. Results

Overall, 166 farms applied to the first 2014–2015 regional call (GPG/2014/996) and 132 farms applied to the call for European funds (EAFRD). Of these, 86 (51.8%) farms implemented mitigation measures with regional funds and only 18 farms (13.2%) with European ones. Concerning regional funding, only 51.1% of available funds (EUR 230,000) were then spent, with an average expenditure of EUR 2674 per intervention. Concerning European funds, the average cost per intervention was EUR 19,996. Notably, the European call for proposals fully recognized labor costs, unlike the regional call for which they were not recognized.

The reasons why farmers renounced funds for mitigation measures showed differences for the two tools used.

For both financing instruments, most of the farmers dropped out because they did not have enough liquidity to anticipate implementing the mitigation measures (41% EAFRD tender vs. 33% regional tender), faced problems with authorization from local administrations (26% EAFRD tender vs. 30% regional tender), did not have enough time compared with the timing of the call (21% EAFRD tender vs. 28% regional tender), or faced other problems (12% EAFRD tender vs. 9% regional tender).

Field technicians collected data from a total sample of 246 farms, and had complete data from interviews and monitoring from 187 of them. Mitigation measures that were realized included fixed metal fences (*n* = 133), mixed metal and electric fences (*n* = 15), semi-permanent electric fences (*n* = 25), mobile electric fences (*n* = 32), guard dogs (*n* = 4), and automated acoustic alarms (*n* = 36). By cumulating predatory events before and after the adoption of mitigation measures, predations decreased from 528 to 35 in the areas made safe, which is a 93.4% decrease in the number of predatory events (Figure 1).

**Figure 2:**
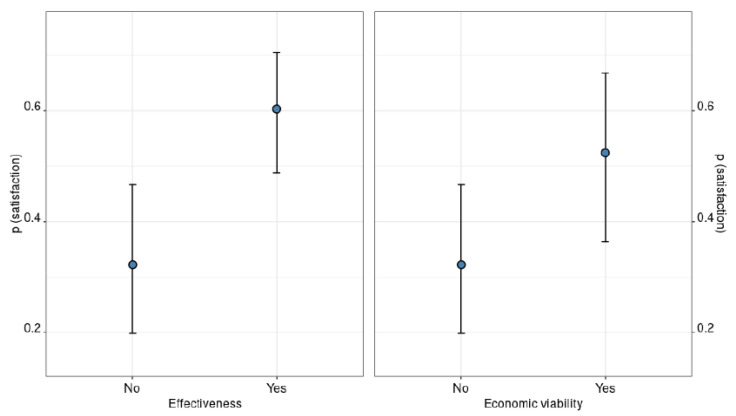
Marginal effect of farmers’ beliefs about the effectiveness and the economic sustainability of mitigation measures over the probability of being satisfied with the adopted measures.

Our best candidate model (AUC = 0.76) explained the satisfaction of farmers with mitigation measures only as a function of two covariates: the perceived effectiveness and the perceived financial viability of the mitigation measure they adopted. Farmers who believed measures to be effective had a 29.2% marginal increase in their probability of being satisfied with them, while farmers who believed measures to be economically feasible had a 21.0% marginal increase in their probability of being satisfied with them (Table 1; Figure 2). Having suffered from livestock predation before or after the implementation of mitigation measures was not associated with farmers’ satisfaction with them.

## 4. Discussion

To the best of our knowledge, this work is amongst the few [6,35] where public data about expenditures for mitigation measures against large carnivores and their damages to livestock have been reported in Europe. By also considering factors associated with abandonment of mitigation measures, as well as by measuring farmers’ satisfaction and perception of mitigation measures, we were able to draw a comprehensive picture of human–carnivore conflicts in Italy and its drivers.

First, the analysis of public expenditures revealed a concerning situation regarding farmers engagement. Only half of available regional funds were used in 2014–2015, and a relatively high proportion of farmers dropped out of the mitigation schemes, renouncing the money, due to serious authorization conflicts. Despite the institutions funding a gamut of prevention measures, offering to cover the costs of the materials or the entire intervention, many farmers were forced to abandon the scheme due to a lack of liquidity to cover initial costs. Animal husbandry in Italy, over the last few decades, has been subjected to a decrease in its financial viability due to a collapse in the price of sheep and cattle milk [36] and in the price of meat [37]. It is not surprising that many farmers currently lack the financial means to cover the implementation of mitigation measures, even when they know that these economic resources will be covered by local authorities within a reasonable time. The magnitude of this phenomenon is truly concerning, with almost one-third of total farmers being in such a fragile situation. This calls for a radical change in policies aimed at mitigating human–carnivore conflicts in Italy. In the short term, as influencing the market price of milk and meat products is unfeasible, regional agencies should focus on policies that provide economic resources to farmers [8] or provide them with a reasonable access to loans. Bureaucratic procedures and expenses for the authorization aspects should be streamlined, since, in the case of small-scale interventions, they may be higher than the costs for the purchase of the material covered by the program. This occurs mainly in areas subject to landscape and environmental constraints where the authorization process for interventions, such as mechanical fences, can be particularly complex and with a timing that is irreconcilable with project deadlines.

The types of interventions that were applied in the context of the two projects, derived from international [38] and national [30] experience and shared with farm managers, guaranteed an excellent response to the predatory problem with a 93.4% (over a period of 4–6 years) lowering of predation on livestock. These data confirm that prevention measures against wolf attacks are a more effective system than post-predation compensation programs [17,39] and demonstrate that active prevention is the only viable path forward for coexistence with large carnivores.

Our analyses also indicate that despite the diffusion of mitigation measures massively reducing predations, the satisfaction of farmers largely depended upon their perception of their implementations, rather than by its real effects. Having suffered predatory events before or after the adoption of mitigation measures was not predictive of farmers’ satisfaction. This might indicate that other factors, such as attitudes toward wolves that originated from prolonged conflicts [15], are far more important, and that short changes in farmer–wolf interactions are unlikely to change preexisting beliefs [40]. In addition, it should be emphasized that the above data concern the space strictly protected by the prevention measure, which often represents a small area compared to that of the farm that can be used for grazing. The interviews showed that although animals are considered safe inside the fences built, the problem exists outside and these fences prevent the use of the property.

However, the finding that farmers’ beliefs about the practical effectiveness of measures and their economic sustainability influence their satisfaction paves the way for future communication initiatives about anti-predatory measures. For local authorities, it would be advantageous to have farmers thinking about these aspects, as it seems that promoting in-depth considerations about them can increase satisfaction with their use and can, in principle, favor their adoption. Of course, these strategies will work only if the procedures for implementing the interventions are simplified and adequate technical assistance is provided for the choice of prevention measures. Through these mechanisms, and through an increased willingness to accept extensions for the implementation of the interventions, it is likely that a larger number of farmers would move toward the path of prevention, with a more effective use of the resources provided by the bodies in charge.

We compared our results with those of other studies that addressed the issue of the validity of prevention measures. For example, Bruns et al. [39] analyzed prevention measures in Germany, quantifying a reduction in predation of 50–100%. Khorozyan and Waltert [22] estimated 100% effectiveness in reducing predation attacks on livestock. Stone et al. [41], in Idaho, compared two areas, one without prevention measures and the other with, and confirmed the clear difference in predation rates in favor of the effectiveness of preventive works. The above data confirm the theory described in this study, and in other studies such as Ambarli [42] and Van Liere et al. [43] conducted in Turkey and Slovenia, which focused on the ineffectiveness of preventive measures. These data show that the success and effectiveness of prevention measures are always closely correlated and interconnected with the environmental, socio-cultural, and geographical context in which they are applied.

The ex post evaluation in this study allowed us to verify the results of the interventions over time. This aspect is of fundamental importance when it is necessary to set up prevention measures, since the effectiveness of some interventions, such as acoustical and light deterrents, as well as guard animals, can erode quickly after one to five months [22]. Interventions such as electric fencing remain highly effective over time, reducing damage for periods ranging from 3 months to 3 years [22].

In choosing the prevention measures to be implemented, it is also necessary to consider the negative impacts of preventive measures on, for example, the throughput of landscapes. Notably, according to local legislation, structural interventions (such as fences) are subject to a rigid and complex authorization process, especially for the landscape aspect that will be significantly transformed/defaced. This is one of the reasons why there are many denials from the beneficiaries of the contributions. Finally, the prevention measures may impact animal production efficiency since animals would find themselves confined to small spaces and with changed habits, which may negatively impact their productivity.

## 5. Conclusions

In relation to the constraints imposed by the European legislation for the compensation of predated animals, there will be a renewed willingness of farmers to implement preventive measures. This willingness coincides with a series of critical issues that must be analyzed and addressed by the bodies in charge to ensure that all farms adopt prevention measures and are compensated in the event of wolf predation. From a technical point of view, the work completed as part of the two projects examined in this study was confirmed to be capable of having a significant impact on livestock predation, but without a simplification of the procedures for requesting assistance, the diffusion of the methods would be limited. Increasing prevention works, technical assistance provided by public bodies, and reimbursements in the event of predation must be considered essential steps in the difficult process of ensuring human–predator coexistence. The lack of even just one of these elements may contribute to a further exacerbation of social tensions, which not only involves humans and wolves, but also the public administration and bodies responsible for managing the problem.

We consider it important to contextualize the results of this work, underlining that (a) in the reference context, the wolf diet is focused on wild animals, guaranteeing wolves a widespread resource on which they may shift their diet; (b) preventive interventions are often accompanied by a change in how the animals are managed, which is easier to achieve in contexts where the predator is present or disappeared for a short time compared with contexts in which the predator has been absent for a long time; and (c) the tailor-made approach and technical assistance are fundamental, so that ad hoc interventions are carried out for each environmental, landscape, and social context. Finally, the multi functionality of the rural environment must always be considered, with all the problems related to tourism, hunting activities, and human activities in general.

## Figures and Tables

**Table 1:** Output of the Bayesian logistic regression. Coefficients are expressed as the logarithm of the odds-ratio.

Variable	Estimate	S.E	95% Credibility Interval
Intercept	−0.75	0.32	−1.39–0.15
Effectiveness	1.18	0.31	0.56–1.79
Economic viability	0.85	0.27	0.31–1.39

## Data Availability

They are available on request from the authors.

## Supplementary Materials

The following are available online at https://www.mdpi.com/article/10.3390/ani11061536/s1, Figure S1: Type of financing; Scheme S1: Constructive schemes of the preventive measures used in the study; Figure S2: Drawings of the different types of fences.
