# Supplementary material for: Use of European Funds and Ex Post Evaluation of Prevention Measures against Wolf Attacks (Canis lupus italicus) in the Emilia-Romagna Region (Italy)
Source: Animals (Basel). 2021 May 25;11(6):1536. doi: 10.3390/ani11061536 (PMC8225206; doi:10.3390/ani11061536)

animals

MDPI

Figure S1. Type of financing. The image shows the different types of financing. **Type 1** provided for fixed metal fences, with underground electro-welded meshes and anti-jump barriers, **type 2** provided for mixed fences, made with an underground electro-welded mesh strip, and two electric cables, **type 3** included multi-wire electric fences with six conductors, and finally **type 4** with kits for electrical networks. Furthermore, the funding could include: **type 5** livestock guard dogs - LGD (including the assistance of a veterinary expert in ethology and behavior) and **type 6** optical-acoustic bollards with timer/PIR activation and random selection of audio files.

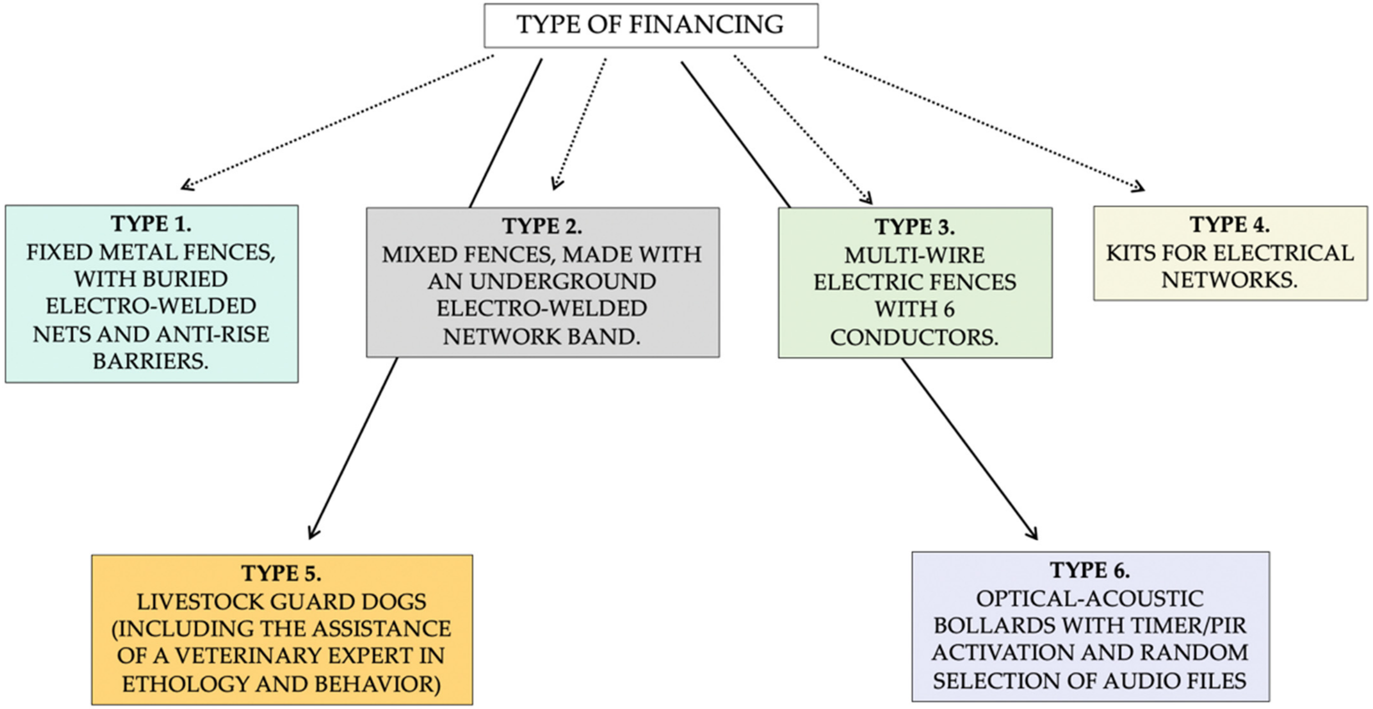

Scheme S1: Constructive schemes of the preventive measures used in the study.

Type 1. Fixed metal fence.

Use: the fence has the purpose of protecting areas where animals are sheltered at night, after milking or in periods at risk of attacks.

Characteristics: realized with electro-welded metal mesh of the building type, buried and folded anti-skip, supported by chestnut poles, with a double-leaf iron gate 3 meters wide with frame and bolt.

Description of materials: electro-welded mesh, preferably in sheets of 300x200 o 400x225, 10x10 mesh 5mm wire or 15x15 wire 6 or heavier, anti-skid folded, buried about 25-30 cm (the sheets are superimposed to allow binding so the useful length becomes about 370 cm instead of 400). Alternatively, mesh sheets of different sizes for equivalent overall height are admissible. Chestnut poles of height 270 cm., Diameter 10/12 cm., Placed at a distance of 370 cm. one from the other, integrated, where necessary, by iron rods.

Type 2. Fixed mixed fencing.

Use: the fence has the purpose of protecting areas where to keep cattle grazing for short periods, ensuring safety for animals and low maintenance.

Characteristics: the realization is foreseen with electro-welded metal mesh from underground building for the lower part and two rows of conductors, connected to an energiser for the upper part. The supports are made up of wooden poles possibly integrated with a building iron rod.

Description of materials: mixed fence with energiser, equipped with an adequate grounding system with at least 2 galvanized ground poles of 1 meter. In the lower part, electro-welded mesh in sheets of 400x225 10x10 mesh 5mm wire or 15x15 wire 6 mm or heavier, cut in half (400x112.5) buried by 30 cm (the sheets are overlapped to allow binding, therefore the useful length becomes 370 cm instead of 400). Alternatively, mesh sheets of different sizes for equivalent overall height are admissible. Chestnut wood posts 200 cm high, 10/12 diameter integrated with iron rod 18-24 mm in diameter and 150 cm high. Warning signs in accordance with the law. Three highly conductive galvanized steel conductors or electrified cable with polyethylene strand and at least 5 copper and / or alloy conductors. Screw insulators are installed only in chestnut poles. In the upper part of the fence, two orders of galvanized steel cable are mounted on screw insulators. The connection between the energiser and the system must be made with a specific super-insulated cable for high voltage.

Type 3. Semi-permanent electrified fence.

Use: the fence has the purpose of protecting medium-sized areas where grazing animals are kept. A greater commitment is required for its efficient maintenance.

Characteristics: the realization is foreseen with chestnut poles 200 cm high (diameter 6-8 or 8-10) fixed at least 35 cm and placed at an adequate distance to follow the profile of the ground. Where difficulties arise, iron poles (12mm construction rod) can be used. Adequate grounding system consisting of at least 2 galvanized poles of at least 1 meter.

Description of materials: high conductivity conductors in galvanized steel or electrified cable with polyethylene strand and at least 5 copper and / or alloy conductors, connected alternately to the energiser or to the earthing system according to the diagram provided by the technician and to the heights indicated in the project. Screw insulators are installed only in chestnut poles, ferrule insulators will be used on iron poles. Warning signs in accordance with the law.

Type 4. Electrified mobile fence.
Use: this type of modular equipment, easily movable, allows the protection of grazing animals on small surfaces.
Characteristics: electrical network modules with a height of about 110 cm, with synthetic poles, to be connected to a battery-powered energiser and photovoltaic panel.
Description of materials: electrified nets with good quality synthetic poles, total height greater than 110 cm, energiser with energy output greater than 2 joules, powered by a rechargeable 12 volts battery and photovoltaic panel of at least 15 watts.
Note for all electrified fences: the electrification system must be sized to ensure the delivery of the following parameters (measured directly on the cables at the point furthest from the energiser): 0.3joules, 3kv.

Type 5. Livestock Guardian Dogs - LGDs.
Use: LGDs ensure the protection of the flock during grazing and shelter. The functionality of this type of prevention is linked to the origin of the animals, to the correct insertion in the stable and their correct education to work. For this reason, only "Maremma sheepdogs" puppies from working lines are recognized, selected for origin and aptitude characteristics. For the choice of puppies and for the insertion of the flock, the costs related to the advice of veterinarians or technicians with specific experience are recognized. The breeder is required to install the necessary information signs on the presence of the dog and to take out an insurance policy for civil liability.

Type 6. Optical-acoustic dissuaders
Use: this type of devices detects the approach of animals and people to the shelter/grazing areas of livestock and exerts a deterrent action by emitting lights and sounds at high volume. The instrument works automatically without operator intervention. It allows the protection of grazing animals or shelters on limited areas and for not too long periods with a very limited commitment on the part of the farmer.
Characteristics: luminous acoustic dissuader that can be activated through PIR sensors and internal timer. The instrument must have the following characteristics and equipment: - construction certified for outdoor use (IP 54) - presence of high-power loudspeaker and LED lights - power supply from rechargeable battery and photovoltaic panel of at least 5 watts - activation by internal PIR sensor, timer programmable and external PIR sensor communicating with the central unit through

a wireless system - emission of random sounds and lights to minimize the addictive phenomenon - presence for each devices of an external wireless PIR sensor resistant to atmospheric agents, a 220 volts battery charger, memory card with digital recordings.

Figure S2. Drawings of the different types of fences.

Type 1. Fixed metal fences, with buried electro-welded nets and anti-rise barriers. External height minimum 165-190 cm and 25 cm underground network.

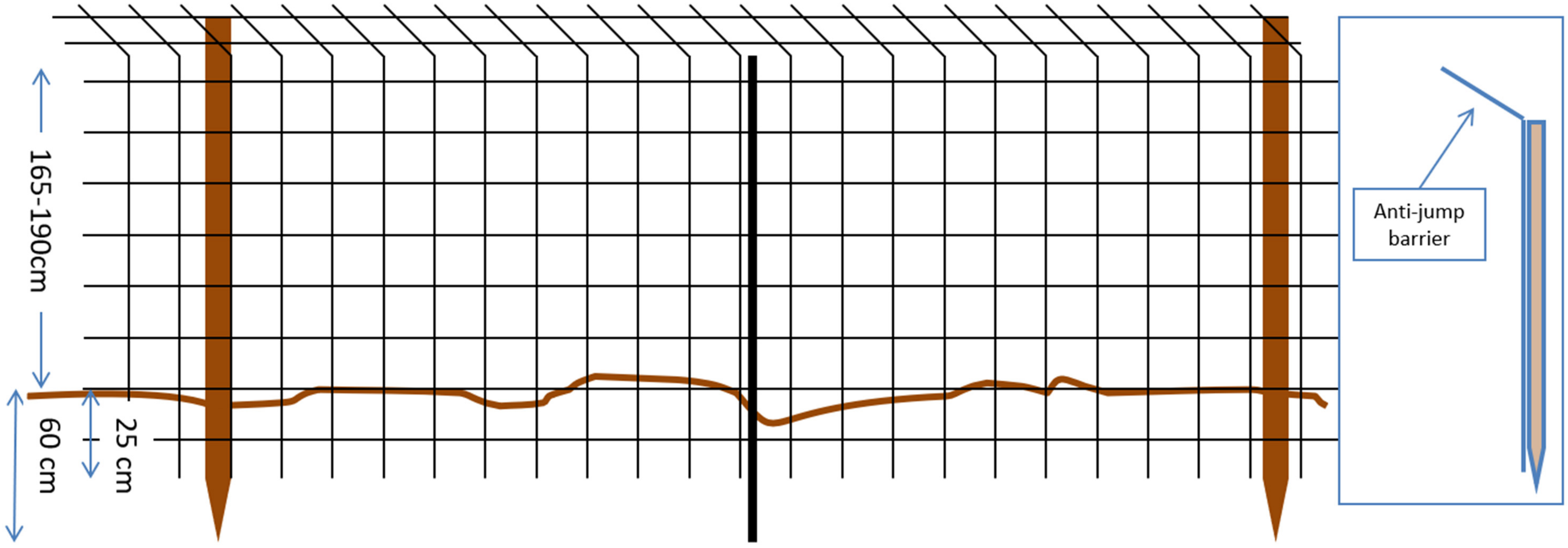

Type 2. Mixed fences, made with an underground electro-welded mesh strip, and two electric cables. External height of 142 cm and 35 cm underground network.

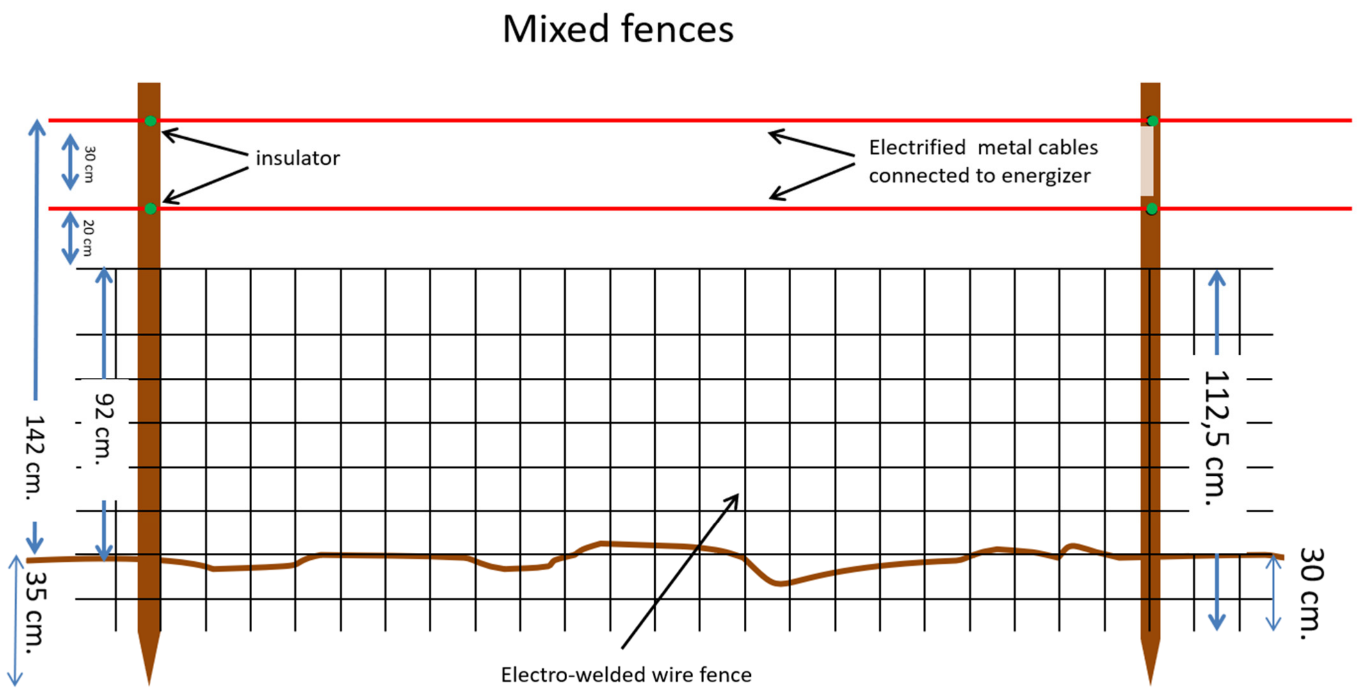

Type 3. Multi-wire electric fences with six conductors**.**

Legend: **(a)** Chestnut pole or other rot-proof essence h 200 cm, tip diameter min. of 8-10 cm driven into the ground of 40 cm, integration if necessary with **(b)** synthetic or iron poles 6 electric cables, height from the ground: 15-35-55-75-100-130. **Minimum total height:** 130 cm. **Cable hight (from the ground):** 15-35-55-75-100-130.

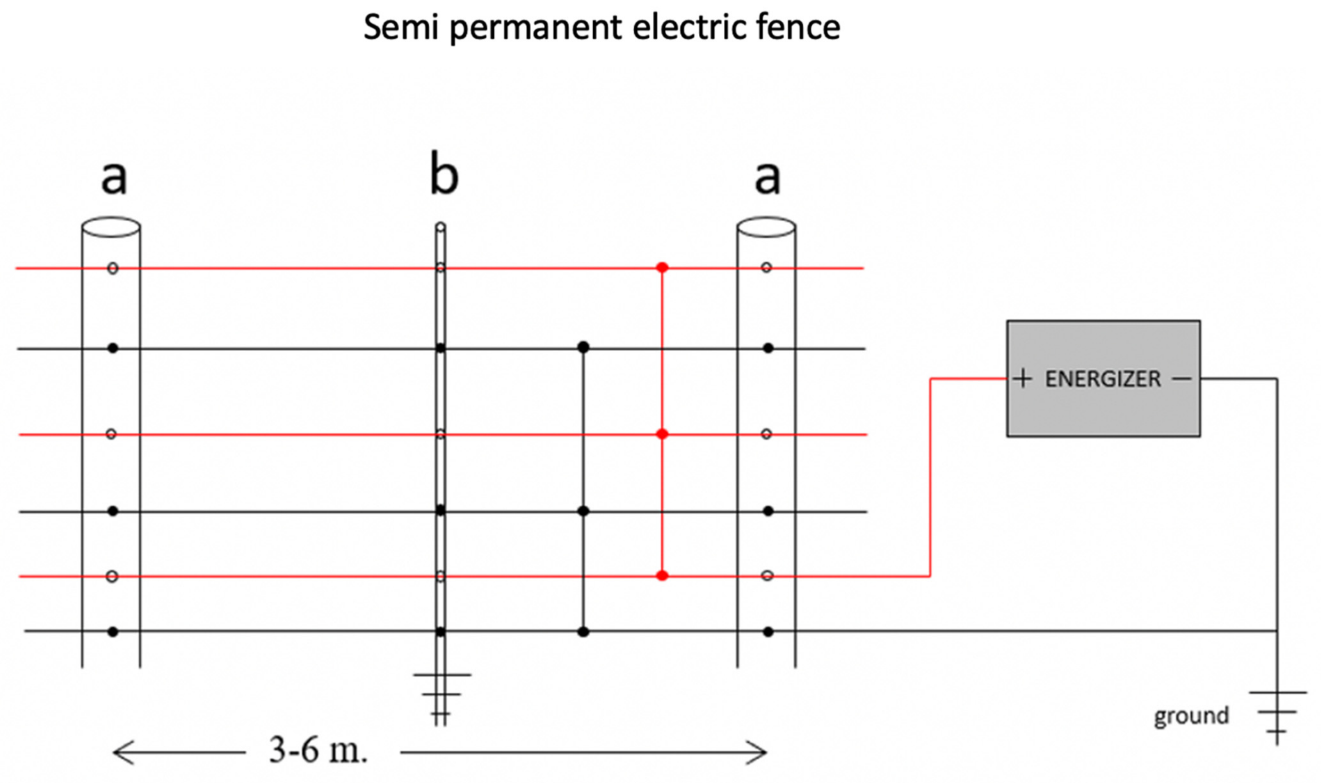

Supplement: Supplementary file 1 [file animals-11-01536-s001.zip › animals-1212252-supplementary.pdf]
